# Solid Pseudopapillary Neoplasm of the Pancreas: A Single-Center Experience of a Rare Neoplasm

**DOI:** 10.7759/cureus.39162

**Published:** 2023-05-17

**Authors:** Sanjay Kumar, Rakesh Kumar Singh, Lajpat Agrawal, Saket Kumar, Tushar Saini, AG Harisankar, Manish Mandal

**Affiliations:** 1 Department of Surgical Gastroenterology and Liver Transplant, Indira Gandhi Institute of Medical Sciences, Patna, IND

**Keywords:** case series, immunohistochemistry, treatment, diagnosis, solid pseudopapillary neoplasm

## Abstract

Background: Solid pseudopapillary neoplasm (SPN) of the pancreas is an extremely rare pancreatic exocrine tumor. The study aims to report our experience with the SPN of the pancreas.

Methods: A retrospective analysis of the prospectively maintained database was carried out of all the cases diagnosed and treated as SPN between January 2019 and January 2023. Patient characteristics including age, gender, clinical presentation, laboratory examinations, imaging features, surgical details, and histopathological and immunohistochemistry details were analyzed.

Results: During this period, eight cases were diagnosed with SPN. All patients were female with a median age of 25.75 years (range 14-55 years). All cases presented with pain in the abdomen, and four patients had a mass per abdomen. In all the cases, contrast-enhanced computed tomography (CECT) abdomen was done for the diagnosis and had preoperative suspicion of the pseudopapillary tumor. In four cases, the tumor was located in the head region, while in four cases, the tumor was in the body and tail of the pancreas. The median size of the tumor was 12 cm (range 3.5-15 cm). Three cases underwent Whipple’s procedure and one patient was unresectable. Two out of four patients with body and tail tumors underwent distal pancreatectomy with splenectomy, one underwent spleen-preserving distal pancreatectomy, and one patient underwent central pancreatectomy.

Conclusion: SPN is a rare neoplasm that primarily affects young women. Clinicopathologic and immunohistochemical features are diagnostic. Surgical resection is generally curative with a good long-term outcome.

## Introduction

Solid-pseudopapillary neoplasm (SPN) is an uncommon exocrine pancreatic tumor that accounts for around 1-3% of all exocrine tumors [1]. These tumors generally occur in young women during the second to fourth decade of life, and they frequently present with nonspecific symptoms such as abdominal pain or discomfort, or the presence of an abdominal mass. A few cases present with back pain, nausea, vomiting, and icterus [2]. The distal pancreas is the most common location for an SPN, followed by the retroperitoneal area [3]. An SPN is composed of poorly cohesive monomorphic epithelial cells forming a miscellaneous growth pattern of solid, pseudopapillary, and pseudocystic structures, along with hemorrhagic degeneration [4]. The origin of these tumors is still a matter of controversy. Surgical resection is considered the best treatment option for SPN, as it is believed to be associated with a malignancy rate of 8-20% [5-7]. Still, no defined pointers can reliably differentiate between benign and malignant varieties of SPN. Although 10-15% of SPN have aggressive behavior, disease-free survival and overall survival rates are much better compared to other pancreatic tumors as long as R0 resection is achieved [8]. In 2010, World Health Organization (WHO) classified it as a low-grade malignant epithelial neoplasm [9].

In this study, we report our experience in the management of SPN of the pancreas in a tertiary-care centre.

## Materials and methods

A retrospective analysis of the prospectively maintained database was carried out of all the cases diagnosed and treated as SPN between January 2019 and January 2023 in a tertiary care center in North India. A total of eight cases having preoperative suspicion of SPN were included in the study. Patient characteristics including age, gender, clinical presentation, laboratory examinations, imaging features, surgical details, and histopathological and immunohistochemistry details were analyzed. All cases underwent routine laboratory examinations, tumor markers, and imaging in the form of either contrast-enhanced computed tomography (CECT) whole abdomen or magnetic resonance imaging (MRI). The surgical procedure was decided by the operating surgeon, based on the location of the tumor. 

Data were entered into Windows 10 Microsoft Excel sheets version 2016 and analyzed using STATA v. 14 software (StataCorp., College Station, TX). Categorical variables like age and tumor size were represented using mean and median.

## Results

Eight female patients with a median age of 25.75 years (14-55 years range) were diagnosed with SPN during this period. All cases presented with abdominal pain, and four had abdominal masses. No patient presented with icterus, gastric outlet obstruction, gastrointestinal bleeding, or pancreatitis.

**Figure 1:**
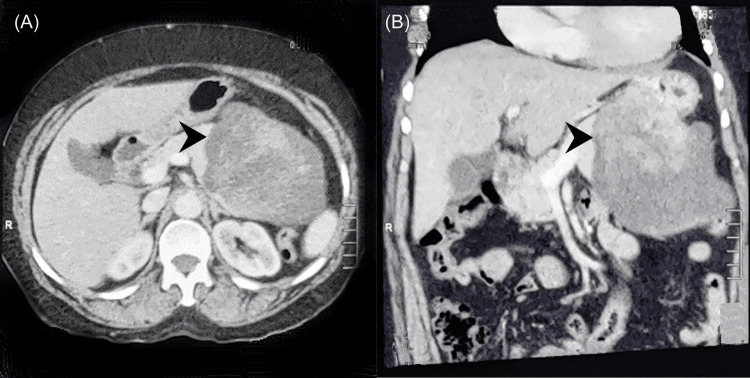
Contrast-enhanced Computed tomography of the abdomen of one patient. (A) axial section (B) coronal section showing large well-defined solid cystic mass with areas of hemorrhage in the tail of the pancreas (arrowhead).

In all the cases CECT abdomen was done for the diagnosis and had preoperative suspicion of the pseudopapillary tumor. Nearly all cases showed the typical features of SPN on CECT i.e. well-defined mixed-density nodules or mass shadows with varying degrees of internal bleeding and cystic degeneration (Figure 1).

All the cases had preoperative tumor markers i.e. CEA and CA 19/9 within the normal range (median CEA and CA 19/9 were 3 ng/ml and 31 U/ml respectively). In four cases the tumor was located in the head region, while in the rest four tumor was in the body and tail of the pancreas. The median diameter of the tumor was 12 cm (range 3.5-15 cm).

Three cases with head lesions underwent Whipple’s procedure and one patient was unresectable due to the involvement of vascular structure, hence ultrasound (USG) guided biopsy was done and the patient was referred for neoadjuvant treatment. Two out of four cases with body and tail tumors underwent distal pancreatectomy with splenectomy, one underwent spleen-preserving distal pancreatectomy, and for one patient laparoscopic central pancreatectomy was done. One patient had an intraoperative superior mesenteric artery injury which was repaired; the patient expired on postoperative Day 2. Two cases had Grade A pancreatic fistula. Patient characteristics are summarized in Table 1.

**Table 1:** Clinicodemographic characteristics of the patients

	Patient 1	Patient 2	Patient 3	Patient 4	Patient 5	Patient 6	Patient 7	Patient 8
Age(years)	15	30	40	19	55	16	14	17
Clinical feature	Pain	Pain, lump	Pain	Pain, lump	Pain	Pain	Pain, lump	Pain, lump
Tumor location	Body, tail	Body, tail	Head	Body, tail	Head	Body	Head	Head
Tumor size	7x5cm	15x12cm	12x10cm	12x10cm	15x12cm	3.5x3.5	15x10	12x10
Surgery	Distal pancreatico-splenectomy	Distal pancreatico-splenectomy	Whipple’s procedure	Distal pancreatectomy	-	Central pancreatectomy	Whipple’s procedure	Whipple’s procedure

**Figure 2:**
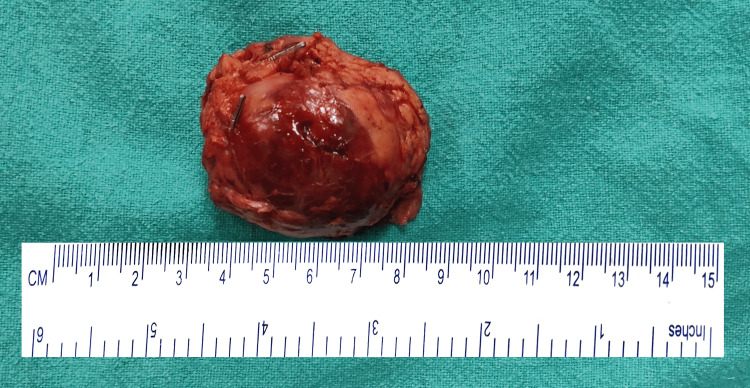
Gross appearance Gross appearance of central pancreatectomy specimen (SPN) showing well-defined lesion of around 3.5 x 3.5 cm. SPN, solid pseudopapillary neoplasm.

**Figure 3:**
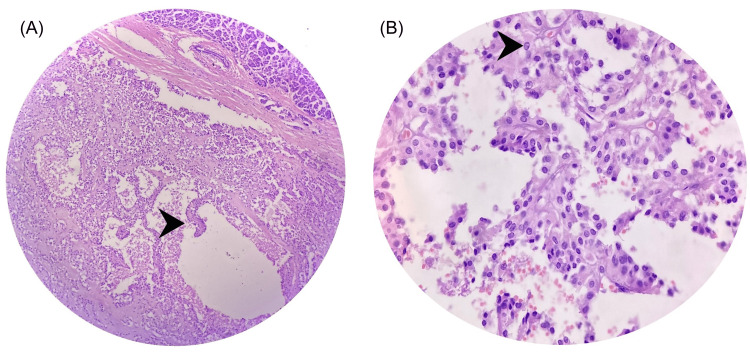
Microscopic appearance (A) H&E (200X) section shows a well-circumscribed lesion showing tumor cells arranged in a solid and pseudopapillary pattern (arrowhead); (B) H&E (400X) section shows individual tumor cells having a moderate amount of eosinophilic to clear cytoplasm. Nuclei have finely textured chromatin with inconspicuous nucleoli (arrowhead).

On gross examination, all lesions were well-circumscribed and had solid cystic components with intermediating areas of hemorrhage (Figure 2). On histopathological examination, the tumor was well circumscribed with adjacent normal pancreatic tissue, and tumor cells were arranged in a solid and pseudopapillary pattern with hyalinization of the stroma. Individual tumor cells had a moderate quantum of eosinophilic to clear cytoplasm. Nuclei had finely textured chromatin with inconspicuous nucleoli (Figure 3). All seven cases had mitosis lower than 2 / 10 HPF. No lymph node metastasis was present in any case.

**Figure 4:**
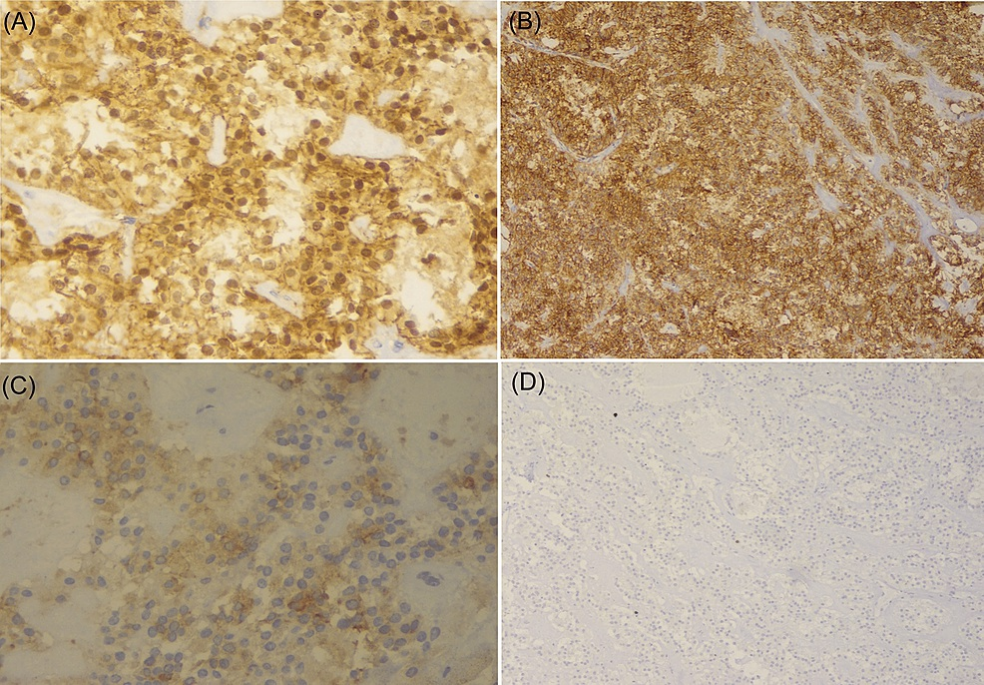
Immunohistochemical staining (A) Beta-catenin is positively expressed in the nucleus and cytoplasm of the tumor cells; (B) tumor cells show nuclear positivity for CD 56; (C) tumor cells show synaptophysin positive; (D) showing low Ki 67 proliferation index

Immunohistochemical analysis was done in five cases. Vimentin, CD10, CD 56, and beta-catenin were positive in all cases (5/5), and synaptophysin was positive in three cases (3/5) (Figure 4). All patients were CEA and chromogranin negative. In one patient, Ki 67 index was 25%, and the rest all the patients had Ki Index lower than or equal to 2%. The immunohistochemistry profiles are summarized in Table 2.

**Table 2:** Immunohistochemistry features CK: cytokeratin; CEA: carcinoembryonic antigen; NSE: neuron-specific enolase; PR: progesterone receptor

	Patient 1	Patient 2	Patient 3	Patient 4	Patient 5
CK	(-)	(+)	(-)	(-)	(-)
Synaptophysin	(-)	(+)	(+)	(-)	(+)
CEA	(-)	(-)	(-)	(-)	(-)
Chromogranin	(-)	(-)	(-)	(-)	(-)
Vimentin	(+)	(+)	(+)	(+)	(+)
NSE	(+)	(-)	(-)	(-)	(+)
CD10	(+)	(+)	(+)	(+)	(+)
CD56	(+)	(+)	(+)	(+)	(+)
PR	(+)	(-)	(-)	(+)	(-)
Ki67	<1%	25%	<1%	2%	2%
Beta-catenin	(+)	(-)	(+)	(+)	(+)

On follow-up, out of eight patients, two patients with head mass were lost to follow-up and one patient expired in the postoperative period. The remaining five patients (three patients of distal pancreatectomy, one patient of central pancreatectomy, and one post-Whipple's procedure) are doing well without any symptoms, recurrence, or distant metastasis. Duration of follow-up varies from six months to four years (median of 16 months).

## Discussion

SPN is veritably rare; in fact, they only constitute about 5% of cystic pancreatic tumors and about 1-2% of exocrine pancreatic tumors [10]. They present largely in life’s second and third decades [11]. This study describes eight cases of solid pseudopapillary neoplasms (SPN) of the pancreas in female patients with a median age of 25.75 years. The origin of solid pseudopapillary tumors remains unclear. These tumors have a ductal epithelial, neuroendocrine, multipotent primordial cell, or indeed an extra-pancreatic genital ridge angle-related cell origin [12].

The clinical feature of the SPN is generally nonspecific. Abdominal pain is the most common symptom, followed by abdominal mass and compression signs due to the tumor. Some cases are asymptomatic, with the tumor detected incidentally by imaging studies or routine physical examination. Generally, there is no evidence of pancreatic insufficiency, abnormal liver function tests, cholestasis, elevated pancreatic enzymes, or an endocrine syndrome. Tumor markers are also generally normal [3,11]. In our series, all cases presented with pain in the abdomen, and four patients had a mass in the abdomen. In all the cases, preoperative tumor markers i.e., CEA and CA 19/9, were normal, reporting that SPN infrequently produces elevated levels of tumor markers. The above findings are consistent with studies done by Kapoor et al. [13] and Yagci et al. [14].

SPN is slightly more common in the tail region but can happen in any part of the pancreas [10]. Grossly, it appears as a large and encapsulated mass, generally well-defined from the remaining pancreas, and invasion of the contiguous organs, similar to the spleen or the duodenal wall, is rare. In our series, four cases had body and tail tumors and four had lesions in the head. In one case, the lesion was infiltrating into the encircling vascular structure.

Abdominal USG and CECT may show a well-defined, complex mass with both solid and cystic components and displacement of nearby structures. There may be calcifications at the periphery of the mass and intravenous contrast enhancement inside the mass suggesting hemorrhagic necrosis [15]. MRI may show certain characteristics similar to hemorrhage, cystic degeneration, or the presence of a capsule which further aids in the differential diagnosis of complex cystic masses within the pancreas [16]. Some studies endorse preoperative endo sonography-guided fine-needle aspiration biopsy for preoperative decision, but this may not be accepted by others because of the possible tumor spread [17,18]. In our series, preoperative CECT was performed on all cases and USG-guided biopsy was done on one case.

Washington et al. showed that the clinical and pathologic features of SPN, including diffuse growth, venous invasion, nuclear pleomorphism, mitotic rate, necrosis, and dedifferentiation, are related to its aggressive behavior or metastatic potential [19]. About 10-15% of SPN have already metastasized at the time of presentation, the most common location for metastasis are the liver, regional lymph nodes, mesentery, omentum, and peritoneum [20]. The disease-free survival and overall survival are much better if R0 resection is achieved, compared to other pancreatic tumors. Hao et al., in their review and metanalysis on a sample of 59 patients with aggressive SPN described a five-year disease-free survival rate of 26.8% and 5- and 10-year overall survival rates of 71.1% and 65.5%, respectively, with recurrence or metastatic rates of up to 69.5% [21]. Although overall and disease-free survival is good even in locally advanced and metastatic patients after curative (i.e. R0) resection, unresectable disease remains the most important predictor of poor survival in all experiences [21]. 

Surgery is the treatment of choice for SPN. It is generally encircled by a pseudo capsule and exhibits benign or low malignant potential. Limited resection with preservation of as much pancreatic tissue as possible is the treatment of choice. Depending on the position of the tumor, distal pancreatectomy with or without splenectomy, pylorus-preserving pancreatoduodenectomy, Whipple operation, or enucleation can be performed. In our series, two cases underwent distal pancreatectomy with splenectomy, one underwent spleen preserving distal pancreatectomy, one underwent central pancreatectomy, and three underwent Whipple’s procedure. Local invasion and metastases are not contraindications for resection. Surgical debulking should be performed for metastases, unlike other pancreatic malignancies. Metastases can be removed with enucleations or lobectomies and some cases with unresectable SPN may also have long-term survival [20]. The overall five-year survival rate with SPN is about 95% [3].

Adjuvant treatment is used only in a small number of cases because of the high resectability of SPN. The role of chemotherapy or chemoradiotherapy in the management of SPN is also not established. In a few studies, adjuvant chemoradiotherapy is given in some unresectable cases with good outcomes [22,23]. Neoadjuvant chemotherapy or chemoradiotherapy has also been successful in a few cases [24-27].

The limitation of the study is the small sample size; also, only five out of seven patients underwent immunohistochemical analysis.

## Conclusions

SPN is a rare pancreatic tumor that usually occurs in young women. It presents as abdominal pain with or without a palpable mass. CECT abdomen is a useful imaging modality for diagnosis. Preoperative tumor markers are usually normal. The treatment of choice is surgical resection, and distal pancreatectomy with or without splenectomy is the most commonly performed procedure. The prognosis is excellent, with a low risk of recurrence and metastasis. The histopathology and immunohistochemistry profile is of paramount importance in the confirmation of diagnosis.
